# Prediction on the Seasonal Behavior of Hydrogen Sulfide Using a Neural Network Model

**DOI:** 10.1100/tsw.2011.95

**Published:** 2011-05-05

**Authors:** Byungwhan Kim, Joogong Lee, Jungyoung Jang, Dongil Han, Ki-Hyun Kim

**Affiliations:** ^1^ Department of Electronic Engineering, Sejong University, Seoul, Republic of Korea; ^2^ Department of Computer Engineering, Sejong University, Seoul, Republic of Korea; ^3^ Department of Environment and Energy, Sejong University, Seoul, Republic of Korea

**Keywords:** model, hydrogen sulfide, generalized regression neural network, radial basis function network, sensitivity analysis, main effect

## Abstract

Models to predict seasonal hydrogen sulfide (H_2_S) concentrations were constructed using neural networks. To this end, two types of generalized regression neural networks and radial basis function networks are considered and optimized. The input data for H_2_S were collected from August 2005 to Fall 2006 from a huge industrial complex located in Ansan City, Korea. Three types of seasonal groupings were prepared and one optimized model is built for each dataset. These optimized models were then used for the analysis of the sensitivity and main effect of the parameters. H_2_S was noted to be very sensitive to rainfall during the spring and summer. In the autumn, its sensitivity showed a strong dependency on wind speed and pressure. Pressure was identified as the most influential parameter during the spring and summer. In the autumn, relative humidity overwhelmingly affected H_2_S. It was noted that H_2_S maintained an inverse relationship with a number of parameters (e.g., radiation, wind speed, or dew-point temperature). In contrast, it exhibited a declining trend with a decrease in pressure. An increase in radiation was likely to decrease during spring and summer, but the opposite trend was predicted for the autumn. The overall results of this study thus suggest that the behavior of H_2_S can be accounted for by a diverse combination of meteorological parameters across seasons.

### INTRODUCTION

Hydrogen sulfide ($H_2S$) is generally recognized as the key component of reduced sulfur compounds (RSCs)[1,2]. It is a colorless, poisonous gas with an odor of rotten eggs. A number of industrial sources, such as natural gas plants, have been identified along with the decomposition from sewage[3]. Exposure to high-level $H_2S$ can cause adverse health effects, such as respiratory stress or olfactory fatigue. There are a variety of meteorological parameters affecting the dispersion and distribution of $H_2S$. They include temperature, relative humidity, dew point, pressure, radiation, direction and speed of wind, etc. A

parametric study was conducted using continuously monitored data or simulations. The Single Point Monitor (SPM) sensor measurements reported an increase in $H_2S$ concentration with the dew-point temperature[4]. A computational fluid dynamics–based study was carried out to predict the leakage and dispersion of $H_2S$ as a function of wind speed[5]. The computation predicted a slow dispersion of $H_2S$ in the leakage jet flow direction and an enhanced danger of exposed individuals in the perpendicular direction to the jet flow. The rate of the increase in $H_2S$ with the wind direction has also been reported[5].

Due to complex interactions between the environmental parameters and $H_2S$, it is very hard to assess the contribution of each parameter with respect to their effect on $H_2S$ behaviors under various environmental conditions. A viable means to accomplish this is to use a nonlinear regression technique named “neural network”. With a high learning and prediction accuracy, the neural network can capture any relationships between multiple parameters and an odorant. Another benefit of the neural network is a quick prediction suitable for a real-time monitoring system. Moreover, it provides an easy adaptation of variations in the environmental parameters by a process of retraining. There have been a number of prediction models constructed for forecasting environmental air pollutants. Neural networks and statistical regression models have been used to predict concentrations of particulate matter (PM 10) with an aerodynamic diameter of <10 μm[6,7,8,9]. The studies[6,7] reported improved predictions of the neural network models over the statistical models. In predicting daily maximum ozone levels as a function of the average air pollutant concentration, the neural network models yielded improved predictions over the statistical linear regression models[10]. A model combining statistical regression and neural network, trained with an extensive dataset including environmental pollutant concentrations and meteorological variables, has been developed to provide improved predictions of ozone concentrations in a lower atmosphere[11]. Neural network models in conjunction with the principal component analysis were built to forecast trends of the three major air pollutants: respirable suspended particulates, nitrogen oxides, and nitrogen dioxide[12]. Concentrations of inorganic airborne pollutants, such as $H_2S$-$SO_2$, NO-$NO_2$—$NO_x$, CO ($CO_2$), and PM 10, have also been predicted using neural networks[13]. Use of neural networks is advantageous in that they can accurately ascertain any relationships between input and output patterns. Other benefits may include a quick response and an easy adaptation to a variation in environmental conditions. However, neural network models are limited in that they are unable to provide a causal relationship in an explicit way.

The level and distribution of air pollutants are considerably influenced by environmental variables. Compared with other earlier works, this work is differentiated in that it proposes seasonal neural network models of $H_2S$ constructed with a large number of environmental variables. Another aspect of the new trial is the use of $H_2S$ models to assess and predict the effect of environmental variables. The $H_2S$ data were collected using an odor monitoring station built in a huge industrial complex located in Ansan City, Korea[14,15]. Two types of neural networks are evaluated as a function of their inherent training factors. Instead of constructing a model for the whole datasets, models are developed for seasonally grouped datasets, and applied to assess the parameters and processes affecting the behavior. The optimized models are thus evaluated by exploring the sensitivity and main effects of meteorological parameters. Furthermore, three-dimensional plots produced from the models are used to investigate seasonal effects of parameters on $H_2S$.

### EXPERIMENTAL DETAILS

#### Site Characteristics

The target study site, the city of Ansan, is well known for the large industrial complex situated on its western side, which deals mainly with metal works, petrochemicals, electronics, and printing. As development proceeded, large apartment buildings were built in the large residential area on the eastern side of the city. Along with this development, traffic consequently expanded. With the air predominantly blowing from the west (industrial complex) towards the east (residential area), complaints grew regarding the industrial processes as the main cause of malodor. In an effort to control odor problems in the city, the concentrations of some major odorant groups, including $H_2S$, were monitored concurrently with such meteorological parameters as temperature, relative humidity, dew point, pressure, wind speed, wind direction, rainfall, and radiation. The analysis of wind direction data and/or its impact on $H_2S$ behavior was treated separately because of its nonscalar properties. The wind rose pattern, when examined using hourly wind direction data, was helpful to understand the common factors affecting the distribution of $H_2S$ at our study site. In addition, detailed evaluation of our datasets has been made in our recent publications to deal with the diurnal and seasonal variations of $H_2S$, along with the analysis of its properties as an offensive odorant[16]. According to this study, $H_2S$ generally exhibited relative enhancement during the night-time period. Although such patterns were seen consistently throughout the seasons, it was most prominent during the summer. In contrast, other RSCs generally exhibited much weaker signals of temporal variations relative to $H_2S$.

### Instrument Settings and Operation

To continuously monitor several target compounds in ambient air, a monitoring station was established at the One-Four park district in Ansan, Korea. The station has been in operation since July 2005, and the concentration levels of $H_2S$ and several other offensive odorants were monitored at hourly intervals from August 2005 to Fall 2006 by an online GC system (with CP-Sill 5CB column pulsed flame photometric detector [PFPD]). In this study, we focus on the analysis of $H_2S$, as it was detected most abundantly during the study period.

Measurements of $H_2S$ in ambient air were made through a combination of an air server (AS) for the collection of air samples and a thermal desorption (TD) unit with a Peltier cooling for preconcentration (and cryofocusing). This combined AS/TD system (Unity/Air Server, Markes, U.K.) was interfaced with a GC system (Model 3800 GC, Varian, U.S.) equipped with PFPD. To acquire the optimum resolution between $H_2S$ and other RSCs, we used a CP-Sill 5CB column (60 m × 0.32 mm, 5.0 µm, Chrompack, Varian) with each cycle to rotate at 60-min intervals (initial oven temperature of 60°C [5-min holding] and a final value of 220°C [ramping at 8°C $min^{-1}$ for 15-min duration]). The measurements of $H_2S$ concentrations were made at hourly intervals. The basic GC conditions were set as follows: cold trap (carbopack B + silicagel) = -15 (low end) to ~280°C (high end); holding time = 5 min; flow path temp = 80°C; outlet split = 5.0 mL $min^{-1}$ (3.2:1 split ratio); carrier gas/column flow ($N_2$) = 2.2 mL $min^{-1}$ (20 psi); detector temperature (PFPD) = 200°C; flow rate: air (1) = 17, air (2) = 10, and $H_2$ = 14 mL $min^{-1}$; square root mode = on; trigger level = 200 mV; PM tube voltage = 50 V; sampling delay = 6 msec; sampling width = 20 msec; gain factor = 20; range = 9; and autozero = no. The basis quality assurance (QA) information of this analysis has been reported elsewhere[16].

## NEURAL NETWORKS

Two types of neural networks were used to build prediction models of $H_2S$. Their fundamentals are briefly introduced.

### Generalized Regression Neural Network

A schematic of the generalized regression neural network (GRNN)[17] is depicted in Fig. 1A. As shown in Fig. 1A, the GRNN consists of four layers, including the input layer, pattern layer, summation layer, and output layer. Each input unit in the input layer corresponds to an individual process parameter. The input layer is fully connected to the second, pattern layer, where each unit represents a training pattern and its output is a measure of the distance of the input pattern from the stored patterns. Each pattern layer

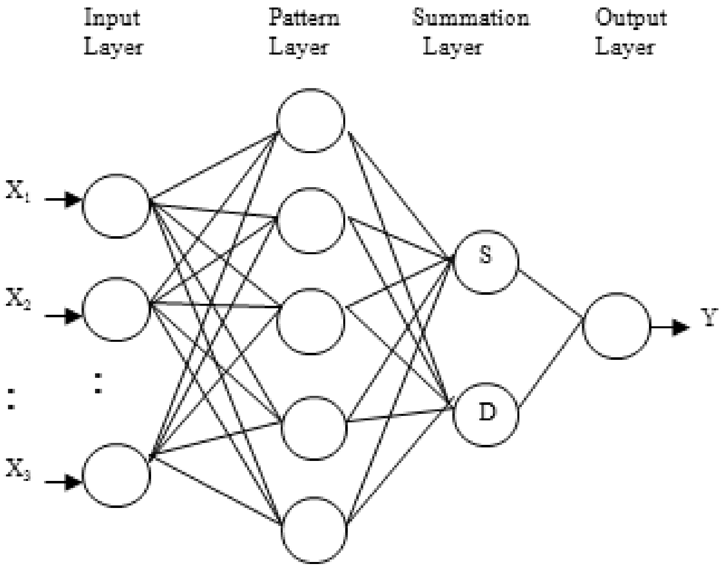

**FIGURE 1A.** Schematic of GRNN.

unit is connected to the two neurons in the summation layer: S- and D-summation neuron. The former computes the sum of the weighted outputs of the pattern layer, while the latter does the unweighted outputs of the pattern neurons. The connection weight between the ith neuron in the pattern layer and the S-summation neuron is $y_i$, the target output value corresponding to the ith input pattern. For the D-summation neuron, the connection weight is unity. The output layer merely divides the output of each S-summation neuron by that of each D-summation neuron, yielding the predicted value to an unknown input vector x as

$$\hat{y}_i(x) = \frac{\sum_{i=1}^{n} y_i \exp[-D(x, x^i)]}{\sum_{i=1}^{n} \exp[-D(x, x^i)]} \quad (1)$$

where n indicates the number of training patterns, and the gaussian D function in Eq. 1 is defined as:

$$D(x, x^i) = \sum_{j=1}^{p} \left(\frac{x_j - x_j^i}{\zeta}\right)^2 \quad (2)$$

where p indicates the number of elements of an input vector. The $x_j$ and $x_j^i$ represent the jth element of x and $x^i$, respectively. The $\zeta$ is generally referred to as the spread, whose optimal value is experimentally determined. It should be noted that in conventional GRNN applications, all spreads for the units in the pattern layer are identical. Despite the simplification of the training process, this may limit the improvement of the GRNN prediction performance. The limitation might be circumvented by adopting a multiparameterization of training factors.

### Radial Basis Function Network

Architecture of the radial basis function network (RBFN)[18] is sketched in Fig. 1B. The RBFN consists of three layers: input, pattern, and output. Each unit in the pattern layer, called pattern unit, calculates its activation using a radial basis function. The activation ($o_j$) is computed as:

$$o_j(x) = \exp[-(x-\mu_j)^2 / \sigma_j^{\,2}] \qquad (3)$$

where $x$ is the input vector, and $\mu_j$ and $\sigma_j$ represent the center and width of a receptive field. The receptive fields are the areas in the input space that activate local pattern units. In Fig. 1B, the RBFN is composed of three receptive fields in the input layer. Each receptive field is composed of a different number of input training patterns. The widths determine the radii of the areas around the centers, in which the activations from the pattern units are significant.

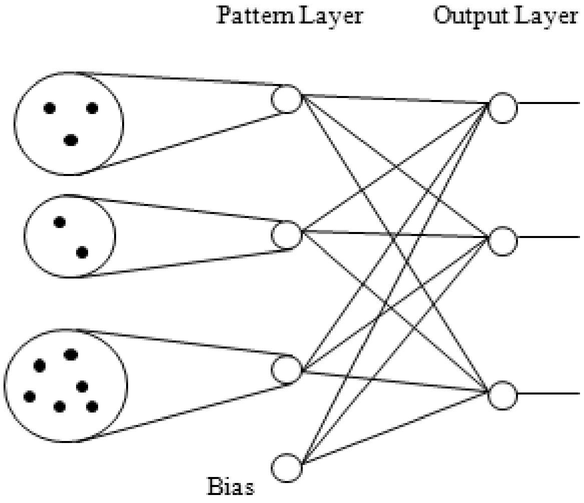

**FIGURE 1B.** Schematic of RBFN.

Training the RBFN consists of two separate stages. In the first stage, the weights between the input and pattern layers are determined given a specific number of pattern units (i.e., clusters). For this, an unsupervised, clustering algorithm called the *k-means algorithm* is typically used. In this algorithm, k training input patterns are first sampled from n training input patterns. These k vectors are regarded as the initial center vectors for the k clusters. For each of the remaining (n-k) training input patterns, Euclidean distance to each center vector is calculated. A training input pattern is then classified into a particular cluster, to which the Euclidean distance is minimized. The center vector of this cluster chosen is subsequently updated by calculating the mean of the center vector and the training input pattern classified. In this way, other training patterns are classified while continuously updating k center vectors. The first stage is completed by assigning each of all the training input patterns to a specific cluster by comparing Euclidean distances with respect to the final updates of the center vectors. In the second stage, the weights between the pattern and output layers are determined in a supervised way. A RBFN error to be minimized is defined as:

$$E = \sum_{j=1}^{m} (y_j - d_j)^2 \qquad (4)$$

where $y_j$ and $d_j$ indicate the prediction from the jth output unit and the actual measurement given to that neuron. The $E$ defined in Eq. 4 is typically minimized by the gradient descent algorithm, and the resulting weight update rule called the *delta rule* is expressed as:

$$W_{ij}(k+1) = W_{ij}(k) + \alpha(y_j - d_j)o_i \quad (5)$$

where $W_{ij}$ represents the connection weight between the ith pattern unit and jth output neuron; $o_i$ indicates the output from the ith pattern unit. The remaining $\alpha$ is the learning rate and it was set to 0.1 in this study. The initial weights are randomly distributed within a predefined range of –1 to 1.

### RESULTS

#### Optimization of Model Prediction Performance

Using the GRNN, the $H_2S$ data are modeled. The range of spreads selected is 0.1 to 1.0. The spread was increased with an increment of 0.1 and one model was constructed at each spread. A total of 10 models were developed. Rather than developing one single model for the whole data, seasonal models were built separately for spring, summer, and autumn data. Each set of seasonal data was divided into three subdatasets for training, validation, and testing the models. The total number of patterns generated from the spring dataset is 3571 and this was divided into the same 1197 patterns comprising each of the three subdatasets. The total patterns of 3516 and 3501 corresponding to the summer and autumn datasets, respectively, were grouped into the three subdatasets of equal size of 1172 and 1167. The performance of the model is quantified by the root-mean square error (RMSE) and the result is shown in Table 1. In Table 1, the training RMSE considerably increases with an increase of the spread from 0.1 to 0.3. In contrast, the corresponding validation and testing errors are decreased. In the remaining range of 0.4–1.0, the training RMSE increases slightly as the spread increases. This is similarly noted for the validation and testing errors. One optimized model with the smallest testing error (0.339) is obtained at 0.3 spread. For the summer and autumn models, the impact of varying the spread is similar in similar ranges. The summer model is optimized at 0.4 spread and the corresponding error is 0.652. For the autumn model, one optimized model is obtained at 0.2 and the corresponding error is 0.361.

**TABLE 1**
**Performance Evaluation of GRNN Models**

| Spread | 0.1 | 0.2 | 0.3 | 0.4 | 0.5 | 0.6 | 0.7 | 0.8 | 0.9 | 1.0 |
|---|---|---|---|---|---|---|---|---|---|---|
| Spring Model | | | | | | | | | | |
| Training | 0.049 | 0.201 | 0.291 | 0.318 | 0.327 | 0.331 | 0.333 | 0.335 | 0.336 | 0.336 |
| Validation | 0.386 | 0.321 | 0.309 | 0.311 | 0.312 | 0.314 | 0.315 | 0.315 | 0.316 | 0.316 |
| Testing | 0.421 | 0.345 | 0.339 | 0.343 | 0.346 | 0.348 | 0.349 | 0.350 | 0.351 | 0.351 |
| Summer Model | | | | | | | | | | |
| Training | 0.119 | 0.327 | 0.455 | 0.510 | 0.530 | 0.537 | 0.541 | 0.544 | 0.545 | 0.547 |
| Validation | 0.628 | 0.540 | 0.523 | 0.523 | 0.526 | 0.528 | 0.530 | 0.532 | 0.533 | 0.534 |
| Testing | 0.704 | 0.661 | 0.653 | 0.652 | 0.653 | 0.654 | 0.655 | 0.656 | 0.656 | 0.657 |
| Autumn Model | | | | | | | | | | |
| Training | 0.091 | 0.234 | 0.294 | 0.316 | 0.325 | 0.329 | 0.331 | 0.333 | 0.334 | 0.335 |
| Validation | 0.428 | 0.409 | 0.405 | 0.405 | 0.406 | 0.407 | 0.409 | 0.410 | 0.411 | 0.412 |
| Testing | 0.369 | 0.361 | 0.372 | 0.376 | 0.378 | 0.379 | 0.381 | 0.382 | 0.383 | 0.384 |

The same $H_2S$ data are modeled using the RBFN. The performance of the RBFN model was optimized by experimentally increasing the number of cluster units from 2 to 10. The results are shown in Table 2. For all RBFN models, the training error appears to slightly increase with an increase in the cluster unit. This is true for other types of error. For the spring model, the smallest testing error (0.348) is obtained for four types of cluster units from 7 to 10. The testing error of an optimized model for the summer data is 0.653 and this is obtained for the cluster units more than 5. For the remaining autumn data, the optimized model occurs for the cluster units of 9 and 10, and the corresponding error is the same at 0.377. As the optimized errors are compared with those of the GRNN models, the latter model shows an improvement of about 2.58 and 4.24% for the spring and autumn data, respectively. Better prediction of the GRNN models over the RBFN models is demonstrated. For the summer data, the performances of the optimized models are comparable. The optimized GRNN models are then used for further analysis.

**TABLE 2**
**Performance Evaluation of RBFN Models**

| Clusters | 2 | 3 | 4 | 5 | 6 | 7 | 8 | 9 | 10 |
|---|---|---|---|---|---|---|---|---|---|
| Spring Model | | | | | | | | | |
| Training | 0.338 | 0.337 | 0.335 | 0.334 | 0.334 | 0.334 | 0.334 | 0.334 | 0.334 |
| Validation | 0.354 | 0.351 | 0.349 | 0.349 | 0.349 | 0.348 | 0.348 | 0.348 | 0.348 |
| Testing | 0.353 | 0.351 | 0.350 | 0.349 | 0.349 | 0.348 | 0.348 | 0.348 | 0.348 |
| Summer Model | | | | | | | | | |
| Training | 0.551 | 0.545 | 0.544 | 0.544 | 0.543 | 0.541 | 0.540 | 0.538 | 0.538 |
| Validation | 0.539 | 0.532 | 0.531 | 0.531 | 0.530 | 0.529 | 0.529 | 0.528 | 0.527 |
| Testing | 0.658 | 0.655 | 0.655 | 0.653 | 0.653 | 0.653 | 0.653 | 0.653 | 0.653 |
| Autumn Model | | | | | | | | | |
| Training | 0.338 | 0.336 | 0.333 | 0.333 | 0.332 | 0.331 | 0.331 | 0.331 | 0.331 |
| Validation | 0.416 | 0.413 | 0.410 | 0.409 | 0.408 | 0.407 | 0.407 | 0.406 | 0.406 |
| Testing | 0.386 | 0.383 | 0.381 | 0.380 | 0.378 | 0.378 | 0.378 | 0.377 | 0.377 |

### Analysis of Parametric Sensitivity and Main Effects

The impact of a parameter can be quantified in terms of its average sensitivity and main effect. We propose an average sensitivity that is calculated for all sampled points of equal distance. This is simply computed by summing all the sensitivities for all the sampled points and subsequently dividing the sum by the number of sampled points. It is noted that the individual sensitivity has already been defined[19]. Here, the distance between two sample points is 0.01 and the total number of sampled points is 200. The average sensitivity is defined as:

$$\text{Average sensitivity} = \left(\sum_{i=1}^{n}\left(\frac{f(x+\Delta x_i)-f(x)}{\Delta x_i}\right)_{\Delta x_i \to 0} \bullet \frac{x_i}{f(x_i)}\right) / \text{n} \quad (6)$$

where $x$ is a vector of environmental parameters maintained at their medium values, $\Delta x_i$ is an incremental change of $x_i$, and $f$ is the functionality that the neural network learns.

The sensitivity calculated from the optimized seasonal models is shown in Fig. 2A–C. The acronyms of T, RH, DP, P, WS, RF, and R are used to represent the temperature, relative humidity, dew-point temperature, pressure, wind speed, rainfall, and radiation, respectively. Fig. 2A reveals that during the spring,

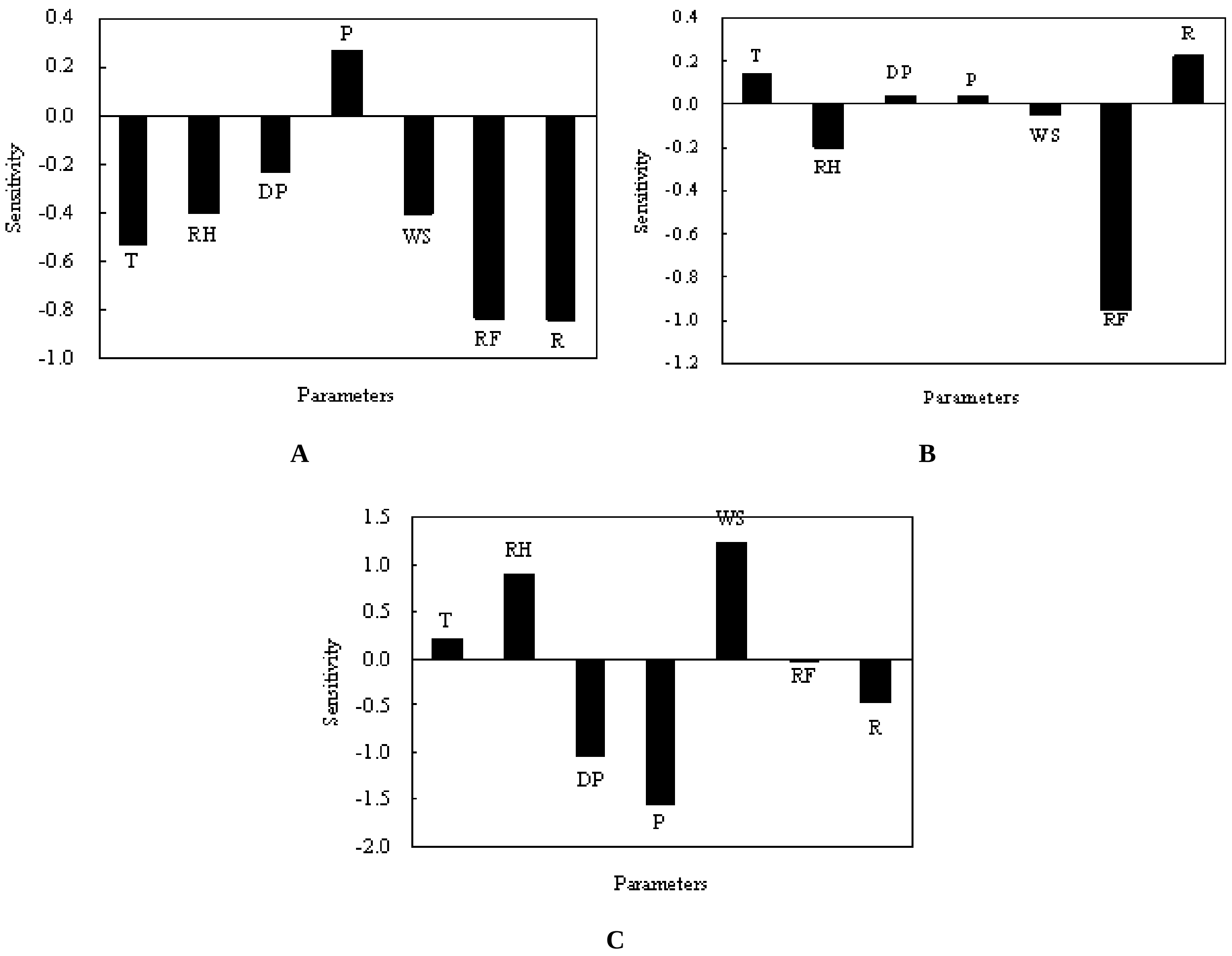

**FIGURE 2.** Sensitivity analysis from the models: (A) spring model, (B) summer model, (C) autumn model.

$H_2S$ is very sensitive to the variation of RF and R. A negative sign indicates that the sensitivity is applicable only where $H_2S$ decreases with an increase in either factor. To the variation of P and DP, $H_2S$ is not sensitive. As illustrated in Fig. 2B, $H_2S$ shows the most pronounced sensitivity to RF during the summer period. An insensitivity to the variation of DP, P, and WS is noted. RF is common in both periods of spring and summer. Fig. 2C shows that $H_2S$ is very sensitive to the variation of WS and P. Interestingly, WS exerts a higher sensitivity as compared to others. Another interesting feature is that they have an opposite sign. In other words, $H_2S$ is sensitive to WS as it is increased and decreased, respectively.

To investigate the main effect of each parameter, the formula expressed in Eq. 6 was slightly modified not to contain the normalization factor. The main effects calculated using the modified formula are shown in Fig. 3A–C. As exhibited in Fig. 3A, $H_2S$ is the most strongly affected by P during the spring period. The parameter P is seen to exert the highest effect during the summer period. This contrasts with its sensitivity. It is noted that the influential parameter P is common for the spring and summer periods. During the autumn, RH is identified as the most influential factor. Its sensitivity is even relatively high.

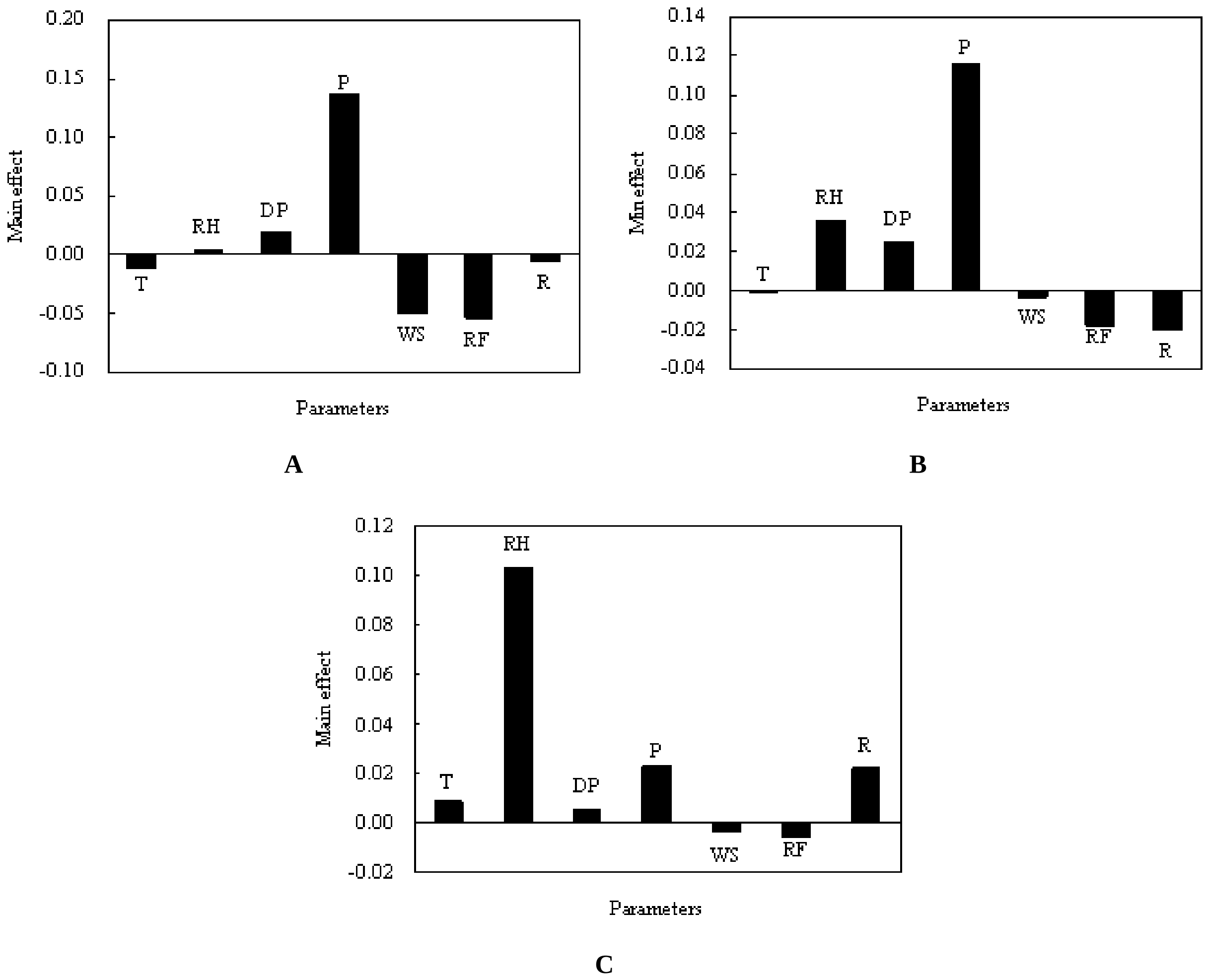

**FIGURE 3**. Analysis of main effects: (A) spring model, (B) summer model, (C) autumn model.

### Model-Based Interpretations

From the optimized seasonal models, main effects on $H_2S$ by the parameters are explored by plotting three-dimensional pictures. The other parameters except the concerned two were set to their medium values of the experimental data. Fig. 4 shows the main effect on $H_2S$ as a function of DP and P during the spring period. An increase in P increases $H_2S$. The positive effect of P was already noted from the main effect analysis. Its effect is highest at a DP of 0°C. As P increases either higher or lower DP than 0°C, the effect becomes smaller. In general, the effect distribution seems to be symmetric relative to 0°C. An increase in $H_2S$ with DP in the range -20–0°C is consistent with the SPM data[4]. Fig. 5 shows the effect of RF and R on $H_2S$. Opposite effects of RF are evident in Fig. 5 depending on its amount. A decrease in $H_2S$ with a decrease in RF is predicted in the first half of the total range. The amount of R is also seen to greatly affect the effect of R. Fig. 5 predicts that in the first half range, varying R causes a little variation in $H_2S$. However, an increase in R in the remaining half range at relatively higher RF drastically decreases $H_2S$. Therefore, Fig. 5 illustrates that the amount of RF plays a significant role in determining the effect of R on $H_2S$. Fig. 6 shows the effect of WS and RF. As WS increases, $H_2S$ decreases and the decrease becomes noticeably large at lower RF. Although there is one turnover point, increasing RF generally decreases $H_2S$ irrespective of WS. This indicates that the effect of RF is not sensitive to WS. This is partly supported by it less sensitivity as shown in Fig. 2A.

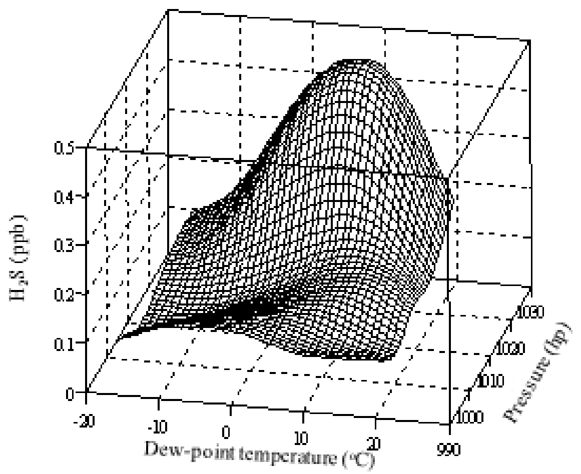

**FIGURE 4.** $H_2S$ concentration during spring as a function of DP and P.

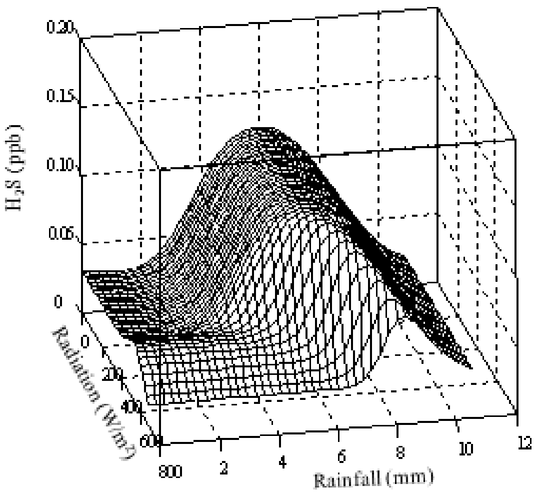

**FIGURE 5.** $H_2S$ concentration during spring as a function of R and RF.

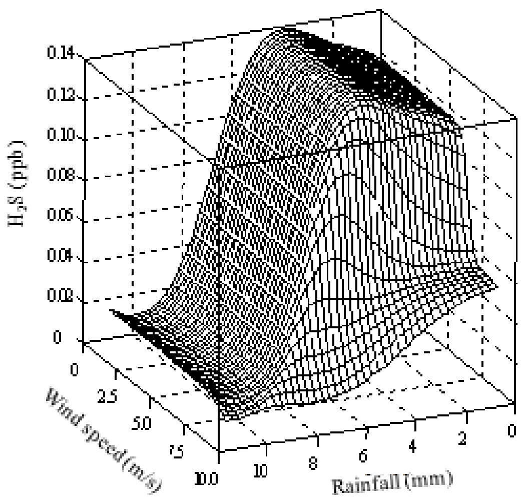

**FIGURE 6.** $H_2S$ concentration during spring as a function of WS and RF.

Fig. 7 shows a plot of $H_2S$ as a function of DP and P produced from the summer model. A decrease in P decreases $H_2S$. The decrease becomes drastic at lower DP. In contrast, $H_2S$ is decreased with an increase in DP. This is similarly predicted from the spring model as shown in Fig. 4. In this sense, both DP and P exert a conflicting impact on $H_2S$. As shown in Fig. 8, an increase of RF in the first half range seems to cause little variation in $H_2S$. In contrast, a further increase in the remaining half range considerably decreases $H_2S$. The effect of R is noticeably large at a lower RF. A similar effect has been predicted from the spring model at a relatively higher RF. In Fig. 9, the effect of RF is very similar to that of the spring model. The effect of increasing WS is seen to decrease $H_2S$ as it increases. This is similarly predicted from the spring model.

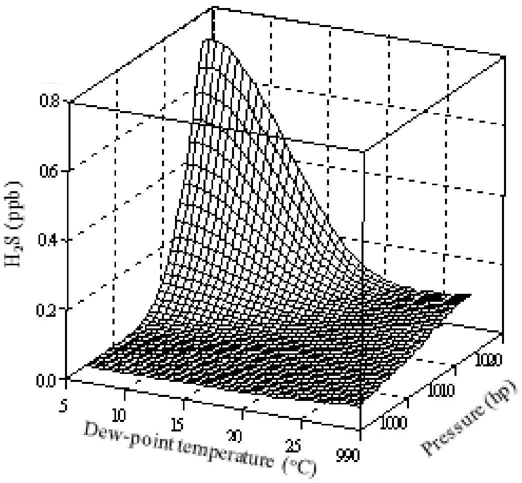

**FIGURE 7.** $H_2S$ concentration during summer as a function of DP and P.

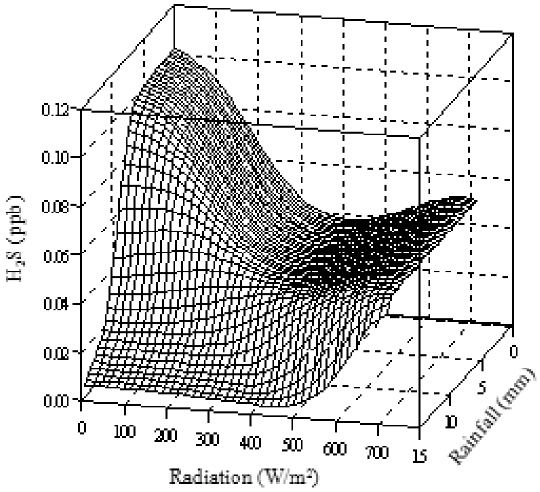

**FIGURE 8.** $H_2S$ concentration during summer as a function of R and RF.

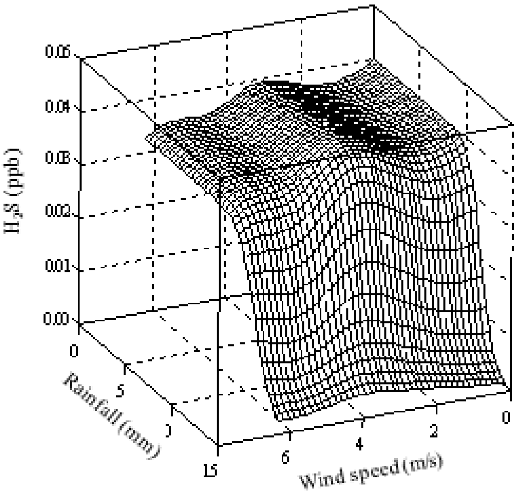

**FIGURE 9.** $H_2S$ concentration during summer as a function of RF and WS.

Plots of $H_2S$ prepared from the autumn model are shown in Figs. 10 and 11. As shown in Fig. 10, the effect of P is similar to those for the spring and summer models. The effect of DP is similar to that for the summer model. With respect to the spring model, a similarity is observed only at the range of positive DPs. For the negative range, the DP effects from the spring and autumn are opposite. In this sense, conflicting DP effects are present only during the spring season. In Fig.11, $H_2S$ is likely to increase with an increase of R. This is similar to that predicted from the summer model at the lower RF, but opposite to that predicted from the spring model at the higher RF.

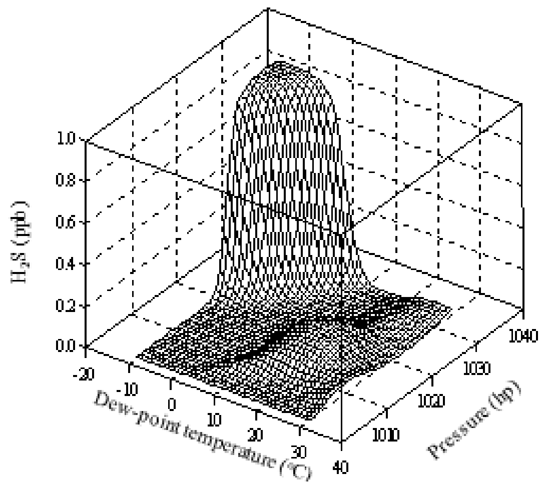

**FIGURE 10.** $H_2S$ concentration during autumn as a function of DP and P.

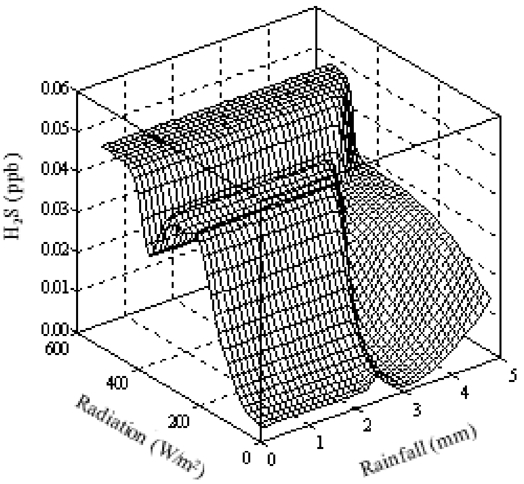

**FIGURE 11.** $H_2S$ concentration during autumn as a function of R and RF.

### CONCLUSIONS

In this study, neural network models were developed and applied to the seasonally divided data groups of a well-known odorant, $H_2S$. Both GRNN and RBFN were optimized as a function of their training factor and the former showed better prediction accuracy. From the optimized GRNN model, analysis of average sensitivity and main effect was conducted. The sensitivity analysis showed different sensitivity of $H_2S$ on the parameters across seasons. During the spring and summer periods, $H_2S$ was found to be the most sensitive to the variation of RF. The main effect analysis revealed that P is the most influential parameter for the period of spring and summer. For the autumn period, the main effect of RH was highest. The optimized models provided useful qualitative information on the parameter effects and they generally showed similar effects without regard to the seasons. $H_2S$ was noted to decrease with an increase either in R, WS, or DP in its positive range as well as with a decrease in P. It is not certain that the reporting tendencies are applicable to any other seasonal data due to the adoption of one year of seasonal data. Nevertheless, each sensor variable is expected to cover a wide range of variables that might occur during each season. From this perspective, the constructed model is likely to provide useful predictions to quantify and predict the effect of the environmental variables. The constructed models can be easily updated by retraining them with newly updated data, thereby providing timely and accurate prediction of environmental characteristics.

### ACKNOWLEDGMENTS

This study was supported by the National Research Foundation of Korea (NRF) Grant funded by the Ministry of Education, Science and Technology (MEST) (No. 2009-0093848) and partly by the Basic Science Research Program through the NRF of Korea (No. 2009-0072846).

## References

1. Kim, K.-H., Choi, Y.-J., Jeon, E.-C., and Sunwoo, Y. (2005) Characterization of malodorous sulfur compounds in landfill gas. *Atmos. Environ.* **39**, 1103–1112.

2. Kim, K.-H., Jeon, E.-C., Choi, Y.-J., and Koo, Y.S. (2006) The emission characteristics and the related malodor intensities of gaseous reduced sulfur compounds (RSC) in a large industrial complex. *Atmos. Environ*. **40**, 4478–4490.
3. Agency for Toxic Substances and Disease Registry (2004) Toxicological Profile for Hydrogen Sulfide. US Department of Health and Human Services, Atlanta.
4. Liang, Y., Xin, H., Hoff, S.J., Richard, T.L., and Kerr, B.J. (2004) Performance of single point monitor in measuring ammonia and hydrogen sulfide gases. *Appl. Eng. Agric*. **20**, 863–872.
5. Zhang, B. and Chen, G.-M. (2009) Hydrogen sulfide dispersion consequences analysis in different wind speeds: a CFD based approach. International Conference on Energy and Environment Technology, Guilin, China, October 16–18. pp. 365–368.
6. Ozkan, O. (2010) Neural network forecast for daily average PM 10 concentrations. *Asian J. Chem*. **22,** 582–588.
7. Chaloulakou, A., Grivas, G., and Spyellis, N. (2003) Neural network and multiple regression models for PM10 prediction in Athens: a comparative assessment. *Air Waste Manage. Assoc*. **53,** 1183–1190.
8. Hooyberghs, J., Mensink, C., Dumont, G., Fierens, F., and Brasseur, O. (2005) A neural network forecast for daily average PM 10 concentrations in Belgium. *Atmos. Environ*. **39**, 3279–3289.
9. Vujic, B.B., Vukmirovic, S.M., Vujic, G.V., Jovicic, N.M., Jovicic, G.R., and Babic, M.J. (2010) Experimental and artificial neural network approach for forecasting of traffic air pollution in urban areas: the case of Subotca., *Therm, Sci.* **14,** S79–S87.
10. Nghiem, L.H. and Oanh, N.T.K. (2009) Comparative analysis of maximum daily ozone levels in urban areas predicted by different statistical models. *SCIENCEASIA* **35,** 276–283.
11. Al-Alawi, S.M., Abdul-Wahab, S.A., and Bakheit, C.S. (2008) Combining principal component regression and artificial neural networks for more accurate predictions of ground-level ozone. *Environ. Model. Softw*. **23,** 396–403.
12. Lu, W.Z., Wang, W.J., Wang, X.K., Yan, S.H., and Lam, J.C. (2004) Potential assessment of a neural network model with PCA/RBF approach for forecasting trends in Mong Kok urban air, Hong Kong. *Environ. Res.* **96(1),** 79–87.
13. Barbes, L., Neagu, C., Melnic, L., Ilie, C., and Velicu, M. (2009) The use of artificial neural network for prediction of some airborne pollutants concentration in urban areas. *Rev. Chim*. **60,** 301–307.
14. Kim, K.-H., Im, M.-S., Jeon, E.-C., Koo, Y.-S., and Youn Y.-H. (2007) An on-line analysis of reduced sulfur gases in the ambient air surrounding a large industrial complex. *Atmos. Environ*. **41**, 3829–3840.
15. Kabir, E. and Kim, K.-H. (2010) An on-line analysis of 7 volatile organic compounds (VOCs) in the ambient air surrounding a large industrial complex. *Atmos. Environ*. **44**, 3492–3502.
16. Susaya, J., Kim, K.-H., Phan, N.-T., and Kim, J.-C. (2011) Assessment of reduced sulfur compounds in ambient air as the potent malodor components in an urban area. *Atmos. Environ*. 10.1016/j.atmosenv.2011.03.051.
17. Specht, D.A. (1991) A general regression neural network. *IEEE Trans. Neural Netw*. **2**, 568–576.
18. Park, J. and Sandberg, I. (1991) Universal approximation using radial-basis-function networks. *Neural Computation* **3**, 246–257.
19. Deif, A. (1983) *Sensitivity Analysis in Linear Systems*. Springer, New York.
